# Adults Do Not Distinguish Action Intentions Based on Movement Kinematics Presented in Naturalistic Settings

**DOI:** 10.3390/brainsci11060821

**Published:** 2021-06-21

**Authors:** Joanna M. Rutkowska, Marlene Meyer, Sabine Hunnius

**Affiliations:** Donders Institute for Brain, Cognition and Behaviour, Radboud University, 6525 GD Nijmegen, The Netherlands; marlene.meyer@donders.ru.nl (M.M.); sabine.hunnius@donders.ru.nl (S.H.)

**Keywords:** intention, intention perception, action prediction, kinematics, movement, EMG, action processing, action understanding, intention understanding

## Abstract

Predicting others’ actions is an essential part of acting in the social world. Action kinematics have been proposed to be a cue about others’ intentions. It is still an open question as to whether adults can use kinematic information in naturalistic settings when presented as a part of a richer visual scene than previously examined. We investigated adults’ intention perceptions from kinematics using naturalistic stimuli in two experiments. In experiment 1, thirty participants watched grasp-to-drink and grasp-to-place movements and identified the movement intention (to drink or to place), whilst their mouth-opening muscle activity was measured with electromyography (EMG) to examine participants’ motor simulation of the observed actions. We found anecdotal evidence that participants could correctly identify the intentions from the action kinematics, although we found no evidence for increased activation of their mylohyoid muscle during the observation of grasp-to-drink compared to grasp-to-place actions. In pre-registered experiment 2, fifty participants completed the same task online. With the increased statistical power, we found strong evidence that participants were not able to discriminate intentions based on movement kinematics. Together, our findings suggest that the role of action kinematics in intention perception is more complex than previously assumed. Although previous research indicates that under certain circumstances observers can perceive and act upon intention-specific kinematic information, perceptual differences in everyday scenes or the observers’ ability to use kinematic information in more naturalistic scenes seems limited.

## 1. Introduction

Recognising others’ intentions and predicting their actions is essential in social interactions [1]. Action kinematics have been proposed as an important cue to perceive others’ intentions [2]. According to this account, one should be able to judge what another person intends to do next just from looking at their movements during the current action step. A large body of literature on intention perception from movement kinematics was recently reviewed by Becchio and colleagues [2]. They suggested that movement kinematics differ depending on the actor’s following action and that observers can perceive this information and use it to identify the actor’s intentions. Reach-to-grasp actions have been frequently utilised in this line of research, since their kinematics depend on the next planned action and thus reflect the intention of the actor. For instance, it has been shown that observers can predict whether a person is going to drink or pour from a bottle by the way they grasp it [3].

This research has traditionally been performed by presenting a simple visual scene: a close-up view on the arm and an object from the side. For instance, Cavallo and colleagues [3] recorded a set of grasp-to-drink and grasp-to-pour movements from that point of view and analysed their kinematic properties. They found that the observers were not able to predict intentions from this stimulus set as a whole, even though the kinematics contained different properties. However, observers performed significantly better than chance when presented with a subset of movements that were chosen as most representative for grasp-to-drink and grasp-to-pour actions. Other studies investigating the role of action kinematics in action prediction used stimulus materials filmed in similar set-ups, but with pre-determined kinematic differences [4,5,6]. Instead of relying on naturally occurring differences in kinematics, Amoruso and colleagues [4] filmed one actor performing the grasping action with different intentions with either precision or whole hand grip. Although this allowed the researchers to pinpoint exactly which kinematic qualities were present in the movements they investigated, it made the stimuli less ecologically valid since the movements had to be carried out in a predetermined manner and could have differed from everyday life situations. Visual aspects such as the point of view might also be especially important for action prediction. Naish and colleagues [7] found that intention perception from action kinematics was the most accurate when the movements were presented from the front, less accurate from a side, and the least accurate from above. However, participants were still not able to predict the following action from grasp kinematics alone. Together, these findings raise questions about the role of movement kinematics in intention perception when it comes to everyday situations.

If kinematics are an important cue for intention perception in everyday life, then they should be also informative when presented as part of a richer visual scene. In the present study, we investigated whether adults can distinguish intentions from kinematic information in a more naturalistic setting. In two experiments, adult participants were presented with videos of actors grasping a cup to drink from it (grasp-to-drink), or to place it somewhere else (grasp-to-place). The videos resemble a typical scene an observer could encounter in everyday life when sitting across from someone at the dinner table. Thereby, the videos are richer and more naturalistic than used in previous research as the cup, the table, and the entire upper body of the actor were visible on the video. The participants’ task was to identify the intention of the actor in a behavioural, forced-choice task with two answers: to drink or to place. We also investigated the role of motor simulation during action observation and intention perception.

Motor simulation of an observed action has been suggested as the mechanism underlying intention perception from action kinematics [6,8]. When one observes someone else executing an action, their own neurons involved in the execution of this action discharge and the electrical activity in the muscles involved in this action increases [8,9]. The mylohyoid muscle responsible for mouth-opening shows anticipatory activation during action execution and observation of grasping actions that lead to opening the mouth, but not during other ones [8], and this muscle activation can be measured using electromyography (EMG) [10]. The present study used EMG to investigate whether the mylohyoid muscle would show anticipatory activity during observation of grasp-to-drink, but not grasp-to-place actions, demonstrating motor simulation during intention perception.

We found that the role of kinematics in intention perception might be limited in naturalistic contexts. The observers were not able to reliably discriminate intentions based on actors’ movement kinematics, and showed no evidence of the motor simulation of the grasp-to-drink action in their mylohyoid muscle.

## 2. Experiment 1

In experiment 1, we hypothesised that participants would be able to identify the intention of the action in the grasp-to-drink and grasp-to-place videos with a higher-than-chance accuracy in the forced-choice task. We also hypothesised that the mylohyoid muscle would show anticipatory activity during the observation of grasp-to-drink but not grasp-to-place videos, providing evidence for motor simulation. We expected the mylohyoid muscle activity to be congruent with participants’ intention perceptions and the correctness of their judgements, so that the mouth-opening muscle activity would be higher when participants evaluated the movement as grasp-to-drink and they were correct than in other cases.

### 2.1. Methods

#### 2.1.1. Participants

Thirty participants from a middle-sized city in the Netherlands took part in the experiment (27 females and 3 males, mean age *M* = 24.3, standard deviation SD = 8.3). They received course credits or a small monetary compensation for their time. The research was approved by the local social science faculty’s research ethics board (ethical approval code: ECSW2016-0905-396), and all participants provided written informed consent. One participant was excluded from the EMG analysis due to too high electrode impedances (>10 kΩ) and another due to technical difficulties.

#### 2.1.2. Stimuli

**Figure 1:**
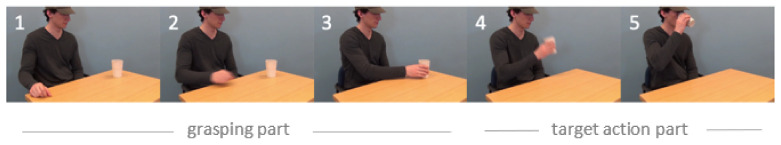
Still frames from an example stimulus video in the drinking condition. Grasp: 1–3, target action (here drink): 4–5.

Stimuli consisted of 64 unique videos of an actor reaching for a cup and either subsequently drinking from it (drink condition, 32 videos) or moving it away (place condition, 32 videos). Figure 1 shows an example drinking sequence. The videos were recorded against a unicolour background in stable lighting conditions, and the actors had colour-matched clothing. The actors all wore the same hat such that their eyes were hidden to the observer. This was done to ensure that the actors did not disclose their intention through their gaze, for example, by looking at the place on the table where they were going to place the cup while grasping. There were 4 different actors (2 female, 2 male), and they used 4 cups that differed in size, shape, and colour. Each actor and each cup featured in the same number of videos in each condition. All videos were cropped to 3 frames (60 ms) before the actor’s hand started moving in the direction of the cup, and to 3 frames (60 ms) after the cup was in the highest position at the mouth (drinking condition) or touched the table (placement condition). The videos had differing lengths (range: 1920–4480 ms, *M*: 2974 ms, SD = 751) as the actors were instructed to perform the actions in a natural manner at their own pace. The videos were subsequently divided into 2 parts: the grasping part and the target action part (see Figure 1). The grasp started at the beginning of the video and ended at the last frame before the cup was lifted from the table (see Video 1 and 2 for example grasp-to-drink stimuli and Video 3 and 4 for example grasp-to-place stimuli in Supplementary Materials). The target action started immediately after and lasted until the end of target action (drink/place). The length of the grasping part ranged between 800 and 1640 ms (*M* = 1101 ms, SD = 203). We found no evidence that they systematically differ between the conditions, as examined with a two-tailed independent samples *t*-test (*t*(31) = 0.482, *p* = 0.633). In addition, two practise trial videos were created in the same way as the stimuli videos (one of each condition, one with a male model, and one with a female model).

#### 2.1.3. Procedure

**Figure 2:**
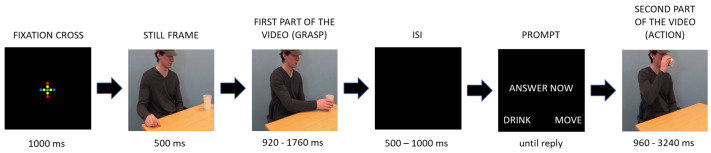
The course of one example trial presented with still frames. The exact timing of each part differed between the stimulus videos (see bottom). ISI—inter-stimulus interval.

Participants were tested individually in a quiet room. First, they gave informed consent. Next, they were seated in a chair in front of a table with a grey button box with two buttons on top. The chair was positioned in front of a screen used to present the stimuli (resolution: 1300 × 800 pixels, refresh frequency: 75 Hz) at a viewing distance of about 60 cm. After the experimenter placed the EMG electrodes under the participant’s chin and on their face and adjusted them if necessary (see Section 2.1.4), they instructed the participant not to speak, to sit still, and to keep their eyes on the screen and their fingers on the buttons of the button box. Then, the experimenter started the EMG recording and the participants started the experimental task (see Figure 2) that was run using Presentation (version 20.0, Neurobehavioral Systems). They were first presented with an instruction screen explaining the task: to watch the first part of the video of an actor grasping a cup, and then decide whether the actor is going to drink from the cup or move it away. They were asked to indicate their judgement by pressing the appropriate button on the button box (right/left, counterbalanced). Before the task started, participants completed two practise trials (one of each condition) to ensure they understood the task.

During the experimental task, the experimenter monitored the participant through a live video feed. Each experimental trial started with the presentation of a fixation cross for 1000 ms (see Figure 2). Before the presentation of the grasp, participants were shown a still first frame of that video for 500 ms, allowing them to familiarise themselves with the scene and to focus on the movement start more easily [8]. After watching the grasp, an inter-stimulus interval between 500 and 1000 ms long followed, and participants were presented with a prompt to answer. After they pressed one of the answer buttons, they were presented with a target action part of the video. Each video was only presented once, resulting in 64 unique trials in total, and the order of stimulus presentation was pseudo-randomised for each participant such that the same actor could not appear more than twice in a row, the same cup could not appear more than twice in a row, and the same action could not appear more than three times in a row. After completing the first half of the trials, the participants were given a short break. They resumed the task whenever they were ready.

#### 2.1.4. Emg Recording and Processing

EMG measurements were taken with Brain Vision Recorder (Brain Products GmbH, Gilching, Germany, 2020). Disposable 4 mm Ambu-Neuroline 700 Ag/AgCl surface electrodes in a bipolar configuration and with a 10 mm inter-electrode distance [11] were used to record muscle activation from the left mylohyoid muscle, over the base of the chin and 1 cm lateral to midline [8]. As required by the setup, the ground electrode was placed on the forehead, below the hairline, and the reference electrode was placed on the mastoid bone behind the ear. A sampling rate of 2500 Hz was used, with a low cut-off of 10 Hz and a high cut-off of 1000 Hz. Before the electrode application, the skin was cleaned using Nuprep Skin Prep Gel, cleanser wipes, and alcohol. To improve electrode impedances, conductive OneStep clear gel was applied onto the already pre-gelled electrodes.

EMG data were first pre-processed with Brain Vision Analyzer 2.1 (Brain Products GmbH). Due to the bipolar set up of the electrodes, the data from one electrode were re-referenced to the other one to obtain a signal for the mylohyoid muscle. Next, a 20–500 Hz bandpass filter, and a 50 Hz notch filter were applied [12], and the data were exported to MATLAB (version 2019a, Mathworks, 2019) using EEGLab [13]. Then, the data were divided into epochs of differing lengths, all of them starting 500 ms before the onset of the freeze frame before the grasp part of the video, and ending 500 ms after the end of the grasp (minimum ISI length; see Figure 2), using ERPlab [14]. Each epoch was visually inspected for excessive movement, and then was full-wave rectified. Trials with excessive movement were excluded from the analyses (0.8% of all trials). Using a custom-made script, the first 500 ms of all remaining epochs was used to calculate the mean pre-stimulus baseline (during fixation cross presentation) for each participant. The first and the last 500 ms of the trial epoch, namely, presentation of still frame and ISI, were removed. The remaining part, encompassing the grasping video presentation only, was stored for subsequent analyses. Due to a differing stimulus length, the EMG signal from each trial epoch was resampled at intervals of 10% of the normalised (to each video) movement time to obtain 10 percentage-based time bins for each trial, for each participant (time bin range: 92–176 ms). Subsequently, each time bin was baseline-corrected to be expressed as a percentage of the mean activation during the pre-stimulus baseline [12].

##### Analysis

Whenever parametric tests were not possible due to a non-normal distribution of the data, their non-parametric counterparts were used. All analyses were conducted in IBM SPSS (version 26, IBM Corporation, Armonk, NY, USA, 2019), unless indicated otherwise.

##### Behavioural Analysis

General accuracy scores were calculated for each participant as a proportion of correct responses out of a total number of responses. To examine whether they were different from chance (0.5), a one-sample one-tailed Wilcoxon signed rank test was performed. In addition, accuracy scores across participants for each trial video were calculated. To investigate a possible learning effect across trials, a Spearman’s rank correlation between trial number and trial accuracy score was conducted.

##### EMG Analysis

The EMG data were averaged across the condition (drink or place) for each participant. A paired-samples *t*-test (one-tailed) was conducted to examine whether the EMG amplitudes differ between the drink and place conditions during the last time bin (just before and at contact time of hand and cup). The last time bin was chosen for analysis based on previous research showing differences between grasp-to-drink and grasp-to-pour actions in the mylohyoid muscle activity to be most distinct at the end of the movement [8,10].

##### Combined EMG and Behavioural Analysis

The EMG data were averaged across four conditions based on participants’ answers (drink/place) and correctness (correct/incorrect). A 2 × 2 repeated measures ANOVA was performed on the data in the last time bin to determine whether participants’ EMG amplitudes reflected their intention perception, measured by the subsequent answer, and its correctness while watching the grasp. This test enabled us to analyse the shared variance related to intention perception between the behavioural and EMG data.

##### Additional EMG Analysis

To establish whether our EMG measure captured the activity of the mylohyoid muscle during action observation, additional analysis of participants’ EMG amplitudes during watching the target action part of the video was conducted. One would expect the EMG activity to be higher during the drinking action than the placement action. Using ERPlab [14], the data from the target action observation were divided into epochs of differing lengths corresponding to each unique length of the video. Then, they were checked for movement artefacts using the moving-window peak-to-peak threshold function with a 100 V threshold. Trials with values exceeding a 100 V threshold and trials whose baselines were previously rejected were excluded from analysis (0.8% of all trials). The data from each epoch were then resampled at intervals of 10% of the normalised movement time to obtain 10 percentage-based time bins (time bin range: 96–324 ms, depending on video length). Subsequently, each time bin was baseline-corrected to be expressed as a percentage of the mean activation during the pre-stimulus baseline [12] and averaged across the conditions for each participant. A one-tailed paired-samples *t*-test was conducted to examine whether the EMG amplitudes differ between the conditions during the last time bin (during the target action—drinking or placing the cup on the desk).

### 2.2. Results

#### 2.2.1. Behavioural Results

**Figure 3:**
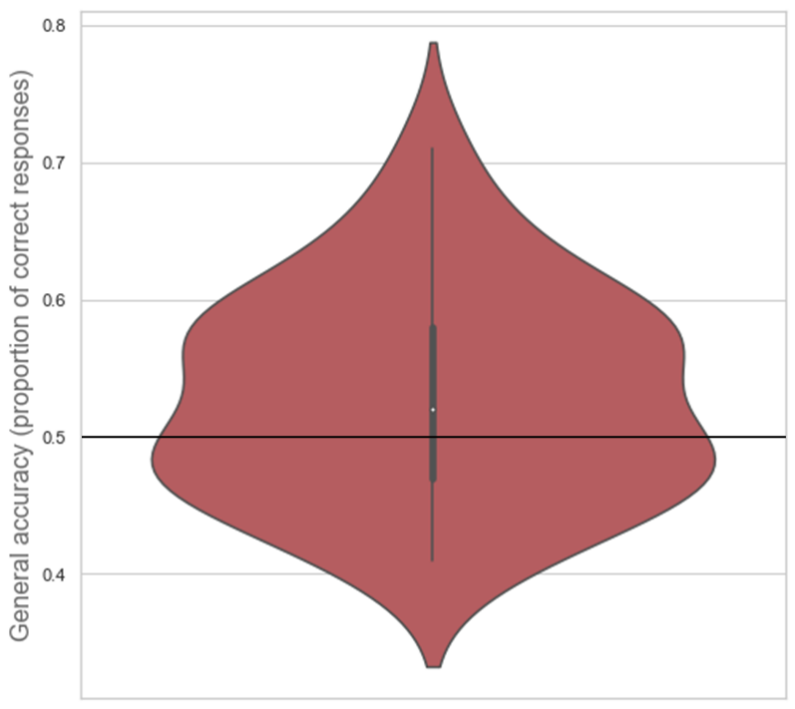
Violin plot of the distribution of general accuracy scores with median (white dot), 1.5× interquartile range (grey vertical line), and chance level (black horizontal line) indicated. Larger surface area indicates more scores at that level.

Participants’ general accuracy ranged between 0.406 and 0.710 for proportion of correct responses (median = 0.508, inter-quartile range IQR = 0.109). The one-sample one-tailed Wilcoxon signed rank test showed that participants’ general accuracy differed significantly from chance level (*Z* = 1.698, *p* = 0.045; see Figure 3).

**Figure 4:**
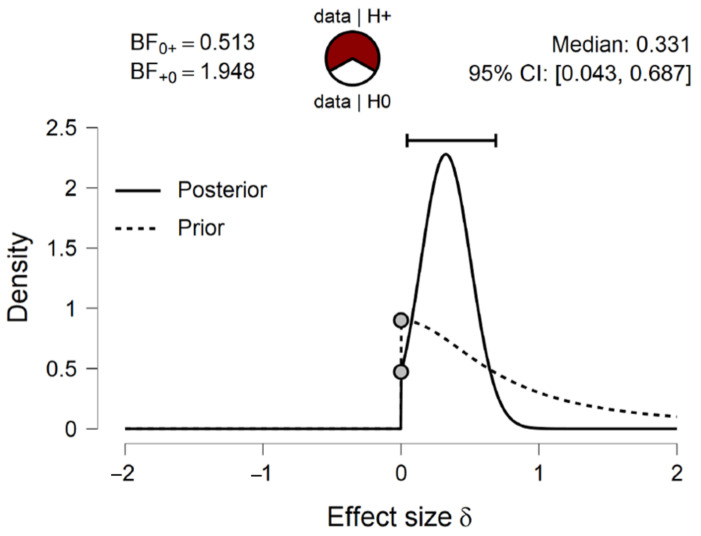
The prior and posterior distribution of the effect size with BF0+ and BF+0, median and 95% Credible Interval (CI) for one-sample one-tailed Bayesian Wilcoxon signed rank test (10,000 samples) on general accuracy score’s difference from chance level (0.5). The distributions indicate that the effect size d is more likely to be larger than 0 under posterior than prior.

Due to the median of participants’ general accuracy being close to 0.5, a one-sample one-tailed Bayesian Wilcoxon signed rank test based on 10,000 samples was conducted in JASP [15] to compare participants’ general accuracy scores with chance level (0.5). A default prior of Cauchy distribution with spread *r* = 1/$\sqrt{2}$ was used, truncated at zero [16]. The test indicated that there was anecdotal evidence that participants performed better than chance (BF+0 = 1.948, Cohen’s *d* = 0.311, *W* = 465.000, $\hat{R}$ = 1.000; see Figure 4).

The Spearman’s rank correlation between trial number and accuracy showed no significant effect ($r_{s}$(62) = 0.094, *p* = 0.462), thus yielding no indication of learning over trials.

#### 2.2.2. Emg Results

**Figure 5:**
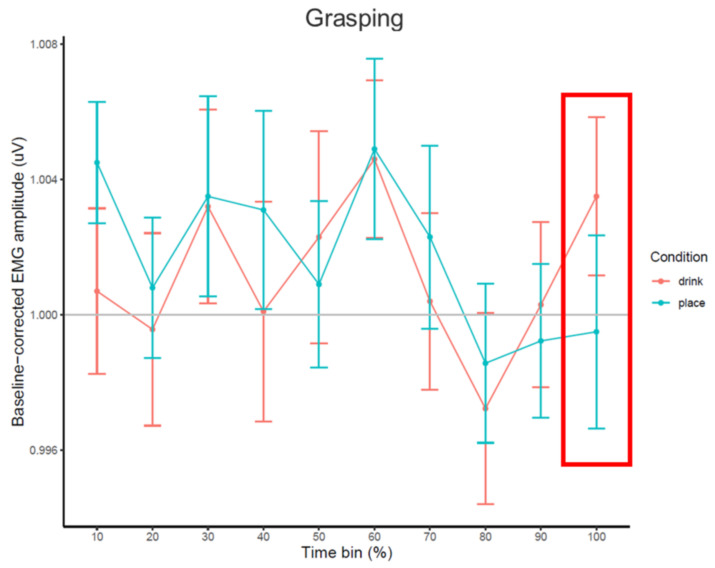
Line chart of average baseline-corrected EMG amplitude (in V) during each percentage time bin of the grasping part for drink and place conditions, with standard error bars (±1 SEM) and a grey line at baseline level (y = 1). Time bin used for statistical analysis is highlighted in the red frame.

The time course of EMG amplitudes based on percentage (%) time bins is presented in Figure 5. The one-tailed paired-samples *t*-test showed no main effect of condition on EMG amplitudes in the last time bin of grasp (*t*(26) = 1.164, *p* = 0.128), so there was no indication that EMG amplitudes differed between the drink (*M* = 1.004, SEM = 0.002) and place conditions (*M* = 1.000, SEM = 0.003).

#### 2.2.3. Combined Emg and Behavioural Results

A 2 × 2 repeated measures ANOVA showed no main effect of answer (drink/place) (*F*(1, 26) = 0.003, *p* = 0.958) or answer correctness (correct/incorrect; *F*(1, 26) = 0.805, *p* = 0.378) and no answer by correctness interaction (*F*(1, 26) = 1.352, *p* = 0.255) on EMG amplitude in the last time bin.

#### 2.2.4. Additional Emg Analysis Results

**Figure 6:**
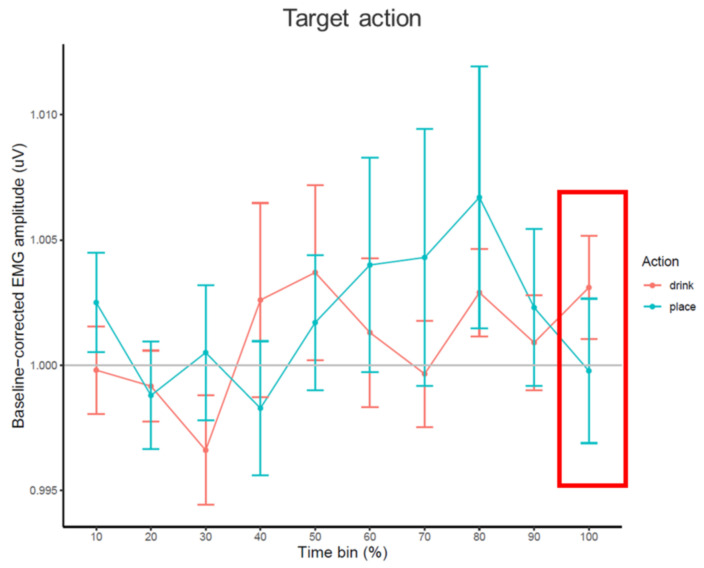
Line chart of average baseline-corrected EMG amplitude (V) during each percentage (%) time bin of the action part of the video for both actions, drink and place with standard error bars (±1 SEM) and a grey line at baseline level (y = 1). Time bin used for statistical analysis in the red frame.

For the time course of EMG amplitudes based on percentage (%) time bins, see Figure 6. The paired-samples *t*-test (one-tailed) showed no main effect of condition on EMG amplitudes (*t*(26) = 1.426, *p* = 0.083), so the amplitudes did not statistically differ between drink (*M* = 1.003, SEM = 0.002) and place conditions (*M* = 1.000, SEM = 0.003) in the last time bin of target action observation.

### 2.3. Discussion Experiment 1

Although the behavioural results suggest that participants could distinguish between intentions from movement kinematics in this study, there was only anecdotal evidence for this effect, and participants’ general accuracy was very low (median = 0.508). The EMG results showed no evidence for a difference in mylohyoid muscle activity between drink and place conditions, suggesting there was no anticipatory activity during the observation of grasp-to-drink actions. Moreover, there was no evidence for a difference in mylohyoid muscle activation that would reflect a different intention perception or its correctness. Additional analysis of the EMG signal from the mylohyoid muscle during target action observation also yielded no significant difference between the conditions. Thus, there was no evidence for the effect of motor simulation in the mylohyoid muscle during grasp or target action observation in this experiment.

## 3. Experiment 2

Due to the mixed results in experiment 1 and only anecdotal evidence for participants’ intention perception from action kinematics, experiment 2 aimed to follow up on the behavioural results to test whether participants can perceive intentions from our naturalistic stimuli with a bigger sample (as determined by power analyses). This allowed us to have a greater statistical power and to provide stronger evidence for or against the effect. Similar to experiment 1, we hypothesised that when presented with a grasp-to-drink or a grasp-to-place video, participants would be able to identify the intention of the action in a behavioural, forced-choice task with a higher-than-chance accuracy. This experiment was pre-registered on the Open Science Framework: https://osf.io/hy38v (accessed on 18 June 2021).

### 3.1. Methods

#### 3.1.1. Participants

Fifty participants from a middle-sized city in the Netherlands took part in the online study (34 females, 16 males, *M* age = 25.3 years, SD = 7.1). They received a small monetary compensation for their time. The research was approved by the local social science faculty’s research ethics board (ethical approval code: ECSW2016-0905-396), and all participants provided informed consent via an online form. The sample size was determined with a power analysis performed with G*Power 3 [17] for a one-tailed one-sample *t*-test with power greater than 0.95, Cohen’s *d* = 0.5, α = 0.05. One participant was excluded from the analysis due to technical errors.

#### 3.1.2. Stimuli

The stimuli were the same 64 unique videos used in experiment 1, with one difference. Only the grasp part of the video was used. All videos started three frames (60 ms) before the actor’s hand started moving in the direction of the cup, and ended at the last frame before the cup was lifted from the table, that is, at the grasp (see Figure 1). There were no practise trial videos used.

#### 3.1.3. Procedure

**Figure 7:**
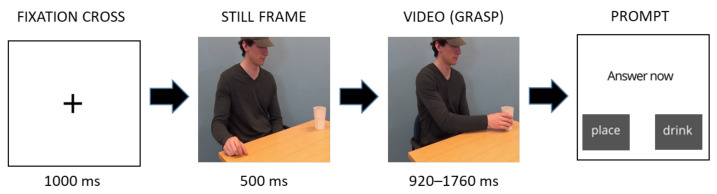
The course of one example trial in experiment 2. The exact duration of the stimuli differed between the stimulus videos (see bottom).

Gorilla Experiment Builder (www.gorilla.sc (accessed on 18 June 2021)) was used to conduct the experiment [18]. Participants could only participate in the experiment online, and they were required to navigate the experiment and answer using their computer mouse. Upon signing up for the study, participants received a link to the informed consent survey. After providing informed consent, they were redirected to the experimental website. They were first asked to indicate their gender and age in years and months. Then, they were presented with the task instructions. They were informed that they would watch short video clips of actors picking up a cup and would have to judge whether the actor was going to drink from the cup or move the cup. They were explicitly instructed to pay attention to the actor’s arm and hand movements. When they were ready, they clicked the start button and started the experiment started. For the course of a trial, see Figure 7. Each experimental trial started with the presentation of a fixation cross for 1000 ms. Then, participants were presented with one of the videos with a still frame inserted at the start for 500 ms (same as in experiment 1). Immediately after the video, participants were presented with a screen with a prompt to answer and two buttons they could click to answer: drink or move. After they pressed one of the buttons, or after 3 s, the trial ended. As in experiment 1, each video was only presented once, resulting in 64 unique trials in total. The order of stimulus presentation was pseudo-randomised into four different sequences, such that the same actor could not appear more than twice in a row, the same cup could not appear more than twice in a row, and the same action could not appear more than three times in a row. Participants were randomly assigned to one of the stimulus presentation sequences. After the experimental task ended, participants were asked to leave a comment if they experienced any problems during the study.

#### 3.1.4. Analysis

As in experiment 1, general accuracy scores were calculated for each participant as a proportion of correct responses out of a total number of responses. As pre-registered, a one-sample one-tailed *t*-test was used to examine whether accuracy scores are greater than chance (0.5). After obtaining a non-significant result from the one-sample *t*-test, a Bayesian one-sample one-tailed *t*-test was conducted to obtain a Bayes factor in order to measure the data’s strength of evidence for the null hypothesis [19]. A default prior of Cauchy distribution with spread *r* = 1/$\sqrt{2}$ was used, truncated at zero [16]. Both tests were conducted in JASP [15].

### 3.2. Results

**Figure 8:**
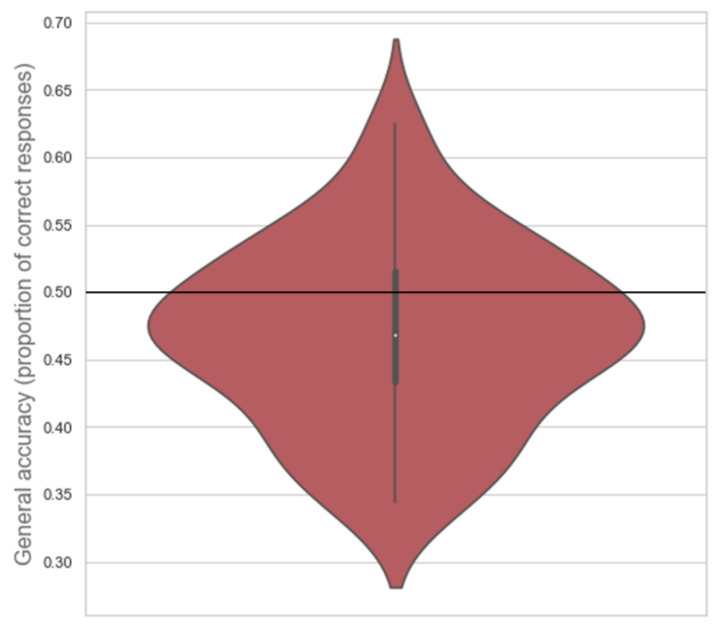
Violin plot of the distribution of general accuracy scores with median (white dot), 1.5× interquartile range (grey vertical line), and chancel level (black horizontal line) indicated. Larger surface area indicates more scores at that level.

Participants’ general accuracy ranged between 0.406 and 0.710 for proportion of correct responses (*M* = 0.469, SEM = 0.010). The one-sample one-tailed *t*-test showed that participants’ general accuracy was not significantly greater than chance (*M* = 0.469, *t*(48) = −3.213, *p* = 0.99; see Figure 8).

**Figure 9:**
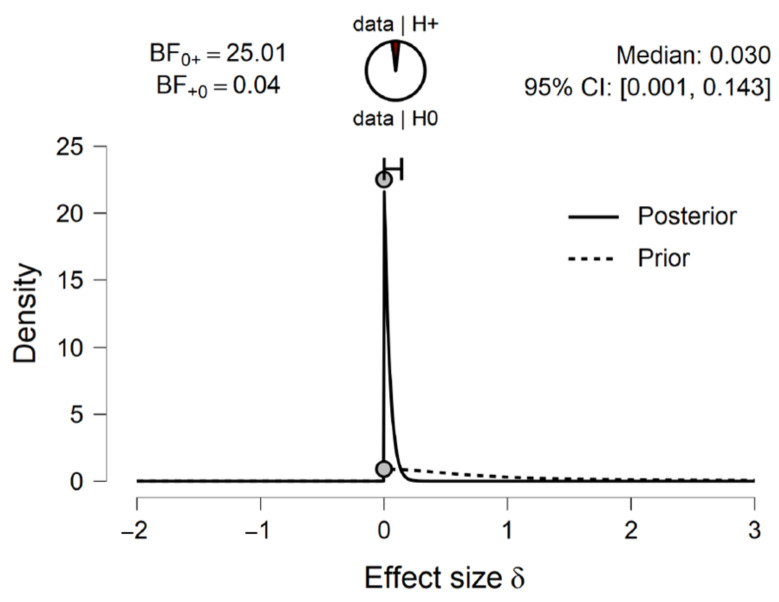
The prior and posterior distribution of the effect size with median and 95% Credible Interval (CI) for the one-sample one-tailed Bayesian *t*-test test on general accuracy score’s difference from chance level (0.5). The distributions indicate that the effect size d is less likely to be larger than 0 under posterior than prior.

The one-sample one-tailed Bayesian *t*-test indicated that there was strong evidence that participants did not perform better than chance (BF0+ = 25.006, *d* = 0.03, 95% CI [0.001, 0.143]; see Figure 9).

### 3.3. Discussion Experiment 2

In contrast to the findings of experiment 1 and despite the increased sample size and the statistical power, we did not find that participants’ accuracy in the intention perception task was better than chance level. On the contrary, experiment 2 found strong support that participants were not able to identify intentions from grasping action kinematics, even though they had been explicitly instructed to do so.

## 4. Discussion

### 4.1. Summary of the Results

Action kinematics have been proposed to work as a cue to perceive others’ intentions [2] with motor simulation of the observed action in the observer working as the mechanism behind it [8]. The aim of this study was to examine whether human adults are able to predict an action by correctly identifying intentions from kinematics in ongoing actions presented in a naturalistic setting. In two experiments, participants watched grasping actions that were either performed with a drinking or placing intention and judged the intention of the grasp in a behavioural forced-choice task. We expected participants to identify the intention (i.e., to drink or place) with a higher-than-chance accuracy based on the grasping movement. In experiment 1, we found only anecdotal evidence that participants performed better than chance, and their performance accuracy was low, indicating that it was hard for participants to distinguish between intentions. Experiment 1 also tested whether the mouth-opening mylohyoid muscle would show anticipatory activity during watching grasp-to-drink, but not grasp-to-place actions, as measured with EMG. Contrary to our predictions, there was no evidence for a difference in the mylohyoid muscle activity between the drink and the place conditions. In addition, there was no relationship between participants’ intention perceptions and their mylohyoid muscle activity during grasp observation. Overall, we found no evidence for motor simulation in the mylohyoid muscle during observation of the grasping actions.

### 4.2. Methodological Limitations

We conducted additional EMG analyses to establish whether our EMG measure captured motor simulation during target action observation (i.e., the drinking and placing actions). We expected higher mylohyoid muscle activity while participants watched the drinking compared to the placing target action. Again, we found no evidence for such an effect. There could be two explanations for these findings: either the stimuli elicited no motor simulation and that was consequently reflected in the EMG signal, or the EMG measure might not have been sensitive enough to pick it up. Although Cattaneo and colleagues [10] also used EMG to investigate intention perception from actions, they used a live stimulus presentation. Since live stimuli elicit a stronger motor simulation response than video stimuli [20,21], our video stimuli might not have elicited a strong enough motor simulation response. Still, in some cases also video stimuli have been shown to elicit measurable motor simulation. For instance, Soriano and colleagues [8] used TMS and EMG combined to measure motor simulation with motor-evoked potentials, which were sensitive enough to register motor simulation while watching video stimuli. Future studies investigating the role of motor simulation in intention perception from kinematics should consider using live stimuli, or could use motor-evoked potentials rather than EMG only with video stimuli. Due to the possible limitations of using video stimuli with EMG, we cannot discern whether there was or was not motor simulation in the mylohyoid muscle during grasping action observation.

It cannot be excluded that the difference in the direction of the evidence between experiments 1 and 2 could be attributed to minor differences in the modality with which we collected data for the intention perception behavioural task. Experiment 1 was conducted in the lab environment, whilst experiment 2 was conducted entirely online. Thus, it is possible that the participants in experiment 2 were less motivated and paid less attention than participants in experiment 1 due to a lack of supervision by the researcher. What speaks against that is that 80% of participants did not miss a single trial by not responding in time, and only one participant missed more than two trials. Therefore, we can be confident that participants paid attention to the experiment for most of its duration. Although we cannot rule out that the participants in experiment 2 were less motivated than in experiment 1, we find that unlikely due to the quick-paced nature of the task and participants’ sustained attention to it.

### 4.3. Possible Explanations

In experiment 2, we tested a bigger sample and explicitly instructed participants to use the movement information of the arm and hand to predict the intention of the actors. Yet, we found that participants did not perform better than chance. Rather, we see strong evidence that participants were not able to perceive intentions from grasping kinematics. Together, the results of the two studies suggest that participants could not reliably identify the intention of a grasping movement presented in a more naturalistic fashion. This stands in contrast with previous studies [3] and suggests that intention prediction from kinematics might be more complex than previously assumed. In the following, we discuss the possibilities that intention-specific kinematic information is less present or salient in more naturalistic actions and that the observers’ ability to use action kinematics to predict intention might be more limited in everyday life than initially thought.

We know from previous research that the visual scene can have an effect on intention perception from movement kinematics [7]. The stimuli used in this study were therefore designed to resemble the visual scene of a realistic social interaction more closely than the stimuli used in previous research [3,4]. In our stimuli, the information in the movement of the whole upper body was available in addition to the grasping kinematics in the movement of the forearm and hand. The stimuli showed different actors and different cups to make the stimuli more naturalistic, since everyday life is full of action-unrelated variability. In addition, each movement was only shown to each participant once. Importantly, that also meant the participants could not identify features idiosyncratic to a single video or actor, but had to use the overarching pattern of the movement kinematics to predict the actors’ intentions. All in all, the richer visual scene and more frontal viewing angle made the stimuli more naturalistic and should have facilitated, rather than prevented, intention prediction from action kinematics.

Since the movement kinematics information in our stimuli was not assessed in detail as it was in Cavallo and colleagues’ [3] study, we ultimately cannot determine whether the findings of our study are a result of the participants’ inability to use the intention information encoded in the movement kinematics, or a lack of such information in the movement kinematics in the current stimuli. If the information in movement kinematics is used as an important cue to others’ intentions in everyday life, however, then that information should be present in our stimuli, as the actors did actually carry out the full actions, that is, they grasped and drank from the cup or placed it on the table, just as they would have in real life. It is possible that the intention information was not always present or not always present to the necessary degree to allow for intention perception. Similarly, in Cavallo and colleagues’ [3] study, participants were not able to perceive intention from grasping kinematics when presented with a random subset of all the movements recorded by the researchers, despite the movements differing in kinematic properties, but were able to do so when presented with only the subset of most representative movements in their categories. The results of both our studies combined suggest that the action kinematics can be an important cue for intention prediction, but their use in everyday life might be limited to a specific extent of kinematic differences between actions, specific viewing conditions, familiarity with the actors’ movements, or to kinematics’ interaction with other contextual information [5,6].

## 5. Conclusions

Although a large body of research demonstrates intention perception from action kinematics (see [2] for a review), this study suggests that action kinematics might play a more complex or limited role in intention perception than previously assumed. When viewing grasp-to-drink and grasp-to-place movements from a naturalistic point of view, participants were not able to reliably distinguish the intention underlying the movement and showed no evidence of motor simulation of the actors’ actions. Thus, the presence of the intention-specific kinematic information in actions or the observer’s ability to use it to identify the actor’s intention might be limited in everyday life. Future studies should investigate these alternatives by measuring both, the amount of information present in actions in naturalistic contexts using motion tracking, as well as observers’ ability to use this information to identify the actor’s intention. Since the present study examined action kinematics in isolation from other cues, it could still have a more prominent role in intention perception in interactions with contextual information.

## Data Availability

All anonymised data are available in the online repository: https://doi.org/10.34973/e573-ab58 (accessed on 18 June 2021).

## Supplementary Materials

The following are available online at https://www.mdpi.com/article/10.3390/brainsci11060821/s1, Video S1: Example of grasp-to-drink stimuli with actor 1, Video S2: Example of grasp-to-drink stimuli with actor 2, Video S3: Example of grasp-to-place stimuli with actor 1, Video S4: Example of grasp-to-place stimuli with actor 2.
